# Display site selection in a ground dwelling bird: the importance of viewshed

**DOI:** 10.1093/beheco/arac112

**Published:** 2022-12-23

**Authors:** Alberto Ucero, Juan C Alonso, Carlos Palacín, Inmaculada Abril-Colón, José M Álvarez-Martínez

**Affiliations:** Departamento de Ecología Evolutiva, Museo Nacional de Ciencias Naturales (CSIC), José Gutiérrez Abascal 2, E-28006 Madrid, Spain; Departamento de Ecología Evolutiva, Museo Nacional de Ciencias Naturales (CSIC), José Gutiérrez Abascal 2, E-28006 Madrid, Spain; Departamento de Ecología Evolutiva, Museo Nacional de Ciencias Naturales (CSIC), José Gutiérrez Abascal 2, E-28006 Madrid, Spain; Departamento de Ecología Evolutiva, Museo Nacional de Ciencias Naturales (CSIC), José Gutiérrez Abascal 2, E-28006 Madrid, Spain; IHCantabria, Instituto de Hidráulica Ambiental de la Universidad de Cantabria. PCTCAN, C/Isabel Torres, 15, 39011, Santander, Spain

**Keywords:** Canarian houbara bustard, *Chlamydotis undulata fuertaventurae*, display site, microhabitat, visibility

## Abstract

We studied the effects of visibility, female and male distribution, microhabitat and distance to human infrastructure on display site selection in a ground-dwelling bird, the Canarian houbara bustard. Using a very high-resolution digital elevation model based on LIDAR technology, and a complete census of the breeding population, we compared 98 display sites with randomly generated sites through generalized linear models. Univariate analyses showed that males displayed at locations that increased their visibility, both at short and long distances. Interestingly, although numbers of females and males around sites did not differ between display and random locations, from display locations males could see more females and males at both distance ranges. The absence of vegetation and stones was also critical as it allowed males to perform display runs on a ground free of obstacles. The amount of trophic resources did not correlate with the selection of the display site itself, though an appropriate vegetation cover seemed to be important at a wider habitat scale. Finally, display sites were farther away than random sites from sources of human disturbance, such as urban nuclei, buildings and tracks. Logistic regression analyses confirmed the importance of viewshed, low stone and vegetation cover, and distance to urban centres and tracks, and model averaging identified short-range visibility and females visible in the long range as the most important visibility variables. These results are compatible with the sexual advertisement and predator avoidance hypotheses. We provide recommendations to ensure an appropriate management of the breeding habitat of this endangered subspecies.

## Teaser

For a male, choosing a suitable display site is critical to effectively transmit his sexual signals, and to do so safe from predators. This is particularly important in terrestrial birds, which have to allow for topographical irregularities and the risks of living on the ground. We show how houbara bustard males display from sites that offer the best viewsheds, and more importantly, maximize the number of visible females, increasing their chances of attracting potential mates.

## INTRODUCTION

In many animal species males perform their territorial songs or sexual displays from specific sites. Since these sites are essential for achieving the two main functions of displays, that is, territory defence and mate attraction, their location should be subject to strong selection (Andersson 1994; Bradbury and Vehrencamp 2011; Kroodsma and Miller 2020). To enhance mate attraction, many lekking birds have developed elaborate visual displays, some of which have probably evolved to reach females at great distances (Johnsgard 1994; Höglund and Alatalo 1995). For example, the white feathers displayed by males of some bustards are visible over 2 or 3 km (Johnsgard 1994; Hellmich 1998, 2003; Jiguet and Bretagnolle 2001; Hallager and Lichtenberg 2007; Ziembicki and Woinarski 2007; Alonso et al. 2010, 2012a). In addition, to optimize visual and vocal signal transmission males usually display from prominent locations and use them repeatedly year after year, which suggests that their selection is related to particular topographic features that maximize long-distance sexual advertisement (Marten and Marler 1977; Hunter 1980; Collins 1981; Petit et al. 1988; Beck and George 2000; Delgado and Penteriani 2007; Alonso et al. 2012a).

The houbara bustard (*Chlamydotis undulata*) is one such species that has developed an elaborate visual and vocal display. Its mating system seems to meet the definition of an exploded lek, with displaying males maintaining inter-individual distances of several hundred meters (Collins 1984; Hellmich 2003; Hingrat et al. 2004, 2008; Hingrat and Saint Jalme 2005; Alonso et al. 2020). Males defend their display sites against intruders and remain faithful to them during the whole breeding season and year after year (Collins 1984; Hellmich 2003; Hingrat et al. 2004, 2008). Male display includes visual and acoustic elements (Gaucher et al. 1996). The visual component consists of rapid circular runs with the head bent backwards, the white breast feathers fully extended and the black throat feathers stretched backwards over the flanks. Each of these runs lasts about 10–20 s, and during the peak display period of the day they can reach frequencies of one run every 1–2 min. Sometimes these runs are in zigzags, figures of eight or straight lines, particularly when there are females nearby (Hinz and Heiss 1989; Hellmich 2003; Cornec 2011, 2015; Cornec et al. 2014, 2015).

In addition to visibility, several other factor may have contributed to display site selection in houbara bustards. Since male display has the dual function of preventing intrusion of neighbor males and attracting females, male and female distribution could be one of such factors. On the other hand, the remarkably fixed use of the same display site year after year strongly suggests that the location of these sites is associated with specific topographic and microhabitat features that could help improving male visibility to potential mates and competitors. The choice of song poles by territorial birds has also been shown to be sensitive to predation risk (Krams 2001; Duncan and Bednekoff 2006; Møller et al. 2006; Parker and Tillin 2006), and it has been suggested that selection of these sites could balance predation risk, mating advantages and feeding benefits (Lima and Dill 1990; Cowlishaw 1997; Heithaus and Dill 2002). In the case of houbara bustard males, their permanent presence and visibility at display sites may attract predators, thus good visibility from these sites is not only important for the transmission of sexual signals, but also for reducing the risk of predation (Bradbury et al. 1989; Wiley 1991; Balmford and Turyaho 1992; Gibson and Bachman 1992; Höglund and Alatalo 1995; Widemo 1997; Boyko 2004; Alonso et al. 2012a). Among behavioral adaptations against predation, one would also expect behaviors to minimize perceived human-derived risks, since humans have become universal predators of many animal species. Indeed, it has been argued that disturbing stimuli could be analogous to predation risk from an evolutionary perspective (Frid and Dill 2002). In addition, urban centres and scattered buildings in the countryside are a source of cats and dogs, potential predators of the houbara bustard (Carrascal et al 2006; Ucero et al 2021). Therefore, distances to human infrastructure should also be important factors affecting display site selection in species living in humanized environments.

Some authors have noted that male houbara bustards display at elevated points from which they have a good field of view and minimize obstacles that could block the transmission of their vocalizations (Hellmich 2003; Hingrat et al. 2008; Cornec 2015; Cornec et al. 2017). Hingrat et al. (2008) conducted a quite comprehensive analysis of display site selection in relation to vegetation and stone cover. On the other hand, other authors have also addressed this issue, but using few variables, very small samples, and ignoring visibility as a relevant factor (Yang 2002; Cuziat et al. 2005; Chammem et al. 2008; Koshkin et al. 2016; Geary et al. 2022). Thus, a rigorous study of the importance of visibility together with other relevant environmental variables has not been carried out to date. Moreover, the importance of the number of females as potential mates and males as competitors, within the viewshed of displaying males, has never been investigated. In the present study we address for the first time a multivariate analysis of the influence of visibility, female and male distribution, microhabitat characteristics and distance to human infrastructure on display site selection of the Canarian subspecies of African houbara bustards (*Chlamydotis undulata fuertaventurae*). Our objective was to investigate which of these factors were most relevant in display site selection. This was only possible by combining the location of display sites, population censuses, detailed topographic analyses using high-resolution digital elevation models (DEM) of the terrain, and accurate microhabitat characterization. To investigate these questions, we conducted a detailed analysis of the viewsheds from display sites. Our sample was large and wide-ranging, including nearly half of all display sites of the species and encompassing its whole distribution in the two islands of the Canarian archipelago holding >95% of the population. All of this allowed for a reliable analysis of display site selection in our study species.

Our main hypothesis was that male houbara bustards select sites with good visibility, with few obstacles that would impede the running display, and that were far from anthropogenic disturbances. Specifically, we tested the following predictions: 1) Males should choose sites with high visibility from which they can observe a large part of their territory and surrounding areas, and benefit from a lower risk of predation (predator avoidance hypothesis; Balmford and Turyaho 1992; Gibson and Bachman 1992; Höglund and Alatalo 1995; Boyko 2004). This would also imply that males should be expected to select sites away from human infrastructure, since humans and associated animals are perceived as predators. 2) Males should display at sites where their visibility to females and other males is higher (sexual advertisement; Andersson 1994; Johnsgard 1994; Bradbury and Vehrencamp 2011). 3) The runways of display sites should be as free as possible of stone cover in order to minimize the risk of injuring the tarsi when performing the running display, and with sparse vegetation to allow for better visibility (Yang 2002; Hingrat et al. 2008). This is important, considering that during the display run the male has its head partially hidden by the display plumage, and that while running it must be also attentive to obstacles and not only to possible predators. 4) If females are attracted by the amount of food in the male territory, the display site should be located in an area with abundant resources (resource defense, or territorial polygyny hypothesis; Ligon 1999; Höglund and Alatalo 1995; Alonso et al. 2012b). Related also to food resources, but from the point of view of the subsistence of the male himself, instead of from the resource defense hypothesis, houbara males display for several hours per day and spend the rest of the day feeding or roosting. In lekking males, the display behavior is energetically demanding (Vehrencamp et al. 1989). Hence, easy access to food resources might be important in display site selection (Yang 2002; Hingrat et al. 2007). Finally, 5) we assessed whether houbara males were faithful to their display sites year after year. Several authors have observed that the same display sites are used every year (Collins 1984; Hellmich 2003; Hingrat et al. 2004, 2008; Hingrat and Saint Jalme 2005), but only some of them used marked birds to check whether these sites were occupied each year by the same individuals (e.g. Hingrat et al. 2004). In any case, a permanent occupation of the same sites, either by different or the same individuals, suggests that these points have certain topographical features that could enhance visibility to potential mates, minimize predation risk, or both (e.g. Alonso et al. 2012a).

## METHODS

### Study area

**Figure 1:**
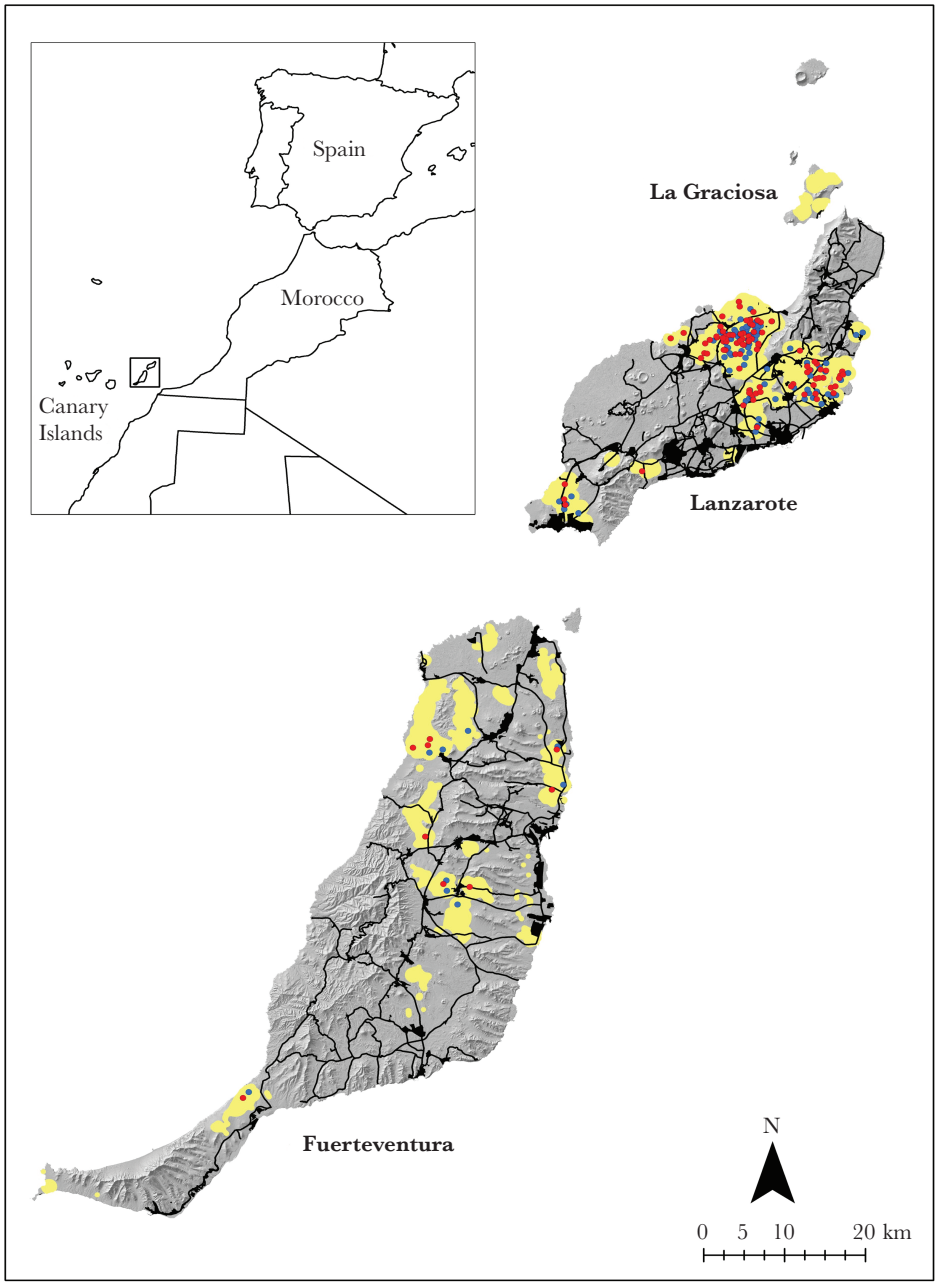
Map showing the distribution of display sites (red dots) and random sites (blue dots) within the distribution range of *Chamydotis undulata fuertaventurae* (yellow-shaded area). Urban centres and roads are shown in black. The distribution range was calculated as a combination of the kernel 95 and a buffer around each bustard location during the breeding population census (see Section 2 for details).

The Canary Islands are located in the Atlantic Ocean, 140 km off the African coast (Figure 1). They consist of eight islands plus some islets, all of volcanic origin. We carried out or study in the two largest easternmost islands, Lanzarote and Fuerteventura (Figure 1), which together hold >95% of the subspecies’ population (Ucero et al. 2021). The two islands are separated by the Bocaina Strait but are connected underwater to form an island unit (Acosta et al. 2003). The strait is only 11 km wide, allowing the movement of houbara bustards between the two islands. The passage of time and the rise in sea level as a result of melting glaciers resulted in the creation of two independent islands (Lambeck et al. 2014). This common past can be seen in their geomorphological and biodiversity characteristics. Both are islands with a low altitudinal relief, predominantly valleys and plains, which explains the low cloud retention that results in an annual rainfall regime of about 110 mm in Lanzarote and 98 mm in Fuerteventura. Due to these characteristics, the biotopes that predominate in Lanzarote and Fuerteventura are sandy formations (*jable*), more or less shrubby, basaltic soils resulting from volcanic eruptions, and stony soils with sparse vegetation.

These areas are characterized by different herbaceous species (grasses, terophytes and herbaceous perennials) and shrubs (*Salsola vermiculata*, *Launaea arborescens*, *Lycium intricatum*, *Suaeda spp.*and *Euphorbia spp*.) and constitute the main habitat of the species. In addition, Canarian houbara bustards use marginal environments, such as edges of lava fields (*malpaíses*) or cultivated areas (Martín and Lorenzo 2001), showing a preference for a mosaic of Launaea shrubland, cultivated land, pasture and fallow land for feeding during the non-breeding season (Ucero et al. 2021; Abril-Colón et al. 2022b), as well as gavias, the traditional cultivated fields in Fuerteventura that are designed to retain rain and runoff water (Medina 1999; Ucero et al. 2021).

### Display and random sites

**Figure 2:**
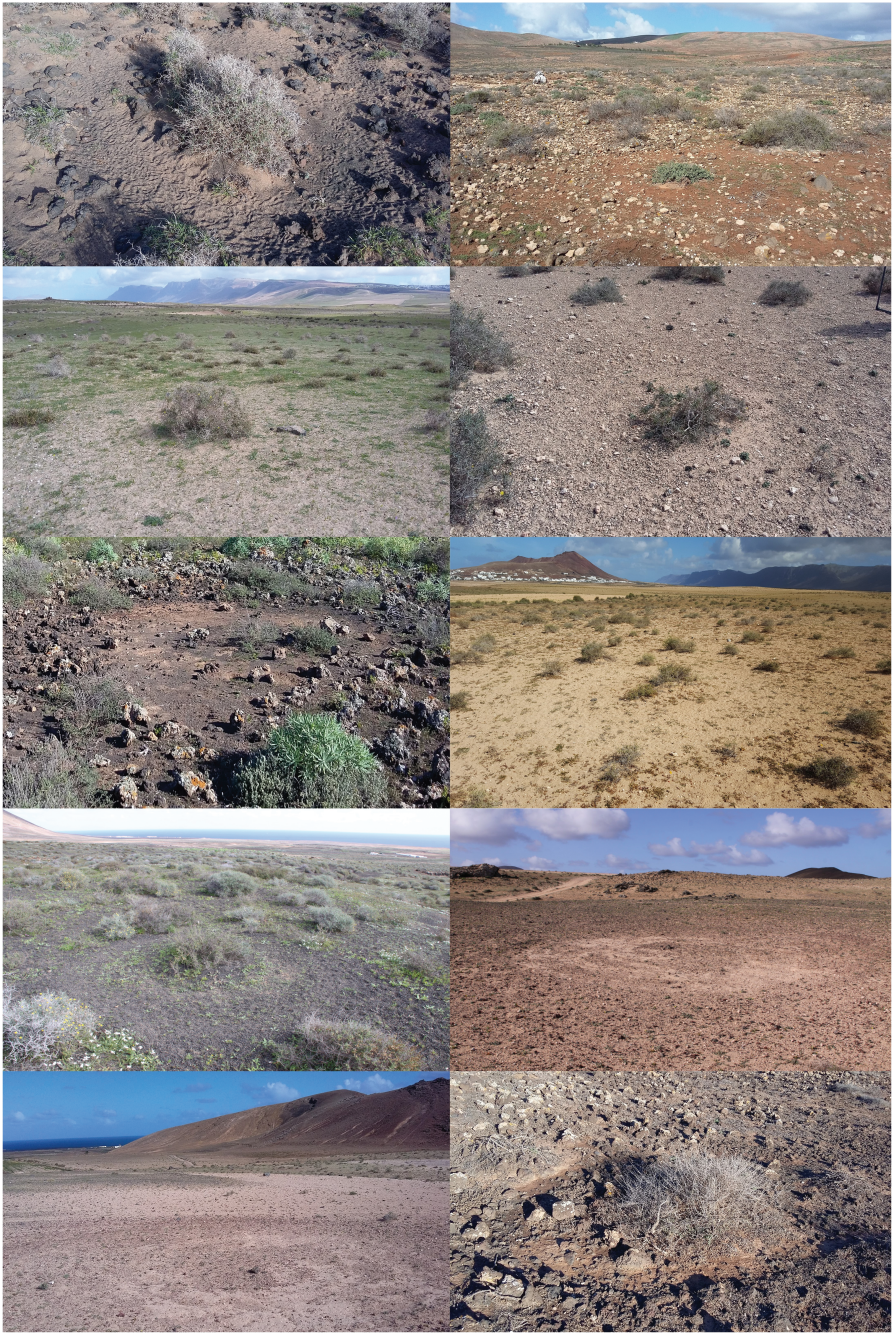
Details of 10 display sites of our sample. The band of ground where the male performs its circular displays can be clearly identified. In some images, the male’s footprints are visible. Photos: Carlos Palacín, Alberto Ucero and Juan Carlos Alonso.

Censuses of the houbara population were conducted between 19 January and 23 February 2018 in Lanzarote (Alonso et al. 2020), and between 7 and 13 January 2020 and 18 and 28 January 2021 in Fuerteventura (Ucero et al. 2021). Details on census techniques can be found in Alonso et al. (2020). In Lanzarote we counted 370 individuals and estimated 440–452 (Alonso et al. 2020), and in Fuerteventura we estimated 85–109 individuals (Ucero et al. 2021). Of these, 196–227 were males (161–182 in Lanzarote, 35–45 in Fuerteventura). Between 2018 and 2021 the display sites of 98 males (25 of which were marked with GSM/GPRS loggers) were visited, a sample that represents ca. half of all display sites (Alonso et al. 2020; Ucero et al. 2021). The sample was evenly distributed within the species’ current range (Figure 1). After observing each male performing circular display runs on the same spot for some time, the exact display site was determined by two observers, one of whom fixed the display site with a telescope, while the other walked to the site where the male had just been watched displaying, both constantly communicating by cell phone to ensure that the correct coordinate of the display site was collected. Upon arrival at the site, the track was often quite visible on the ground and remains of droppings and feathers were found, confirming that the site had been used by the male very frequently (Figure 2). Subsequently, several visits were made to all display sites to confirm that they had been used permanently throughout the breeding season. The repeated use of the same site within and between years was also confirmed using tagged males (17 males in 2018–19, 20 males in 2019–20, 16 of them with data in both years). The 3D-graphs provided by the accelerometers (ACC) with which the transmitters were equipped enabled us to recognize several behaviors including display runs—performed by marked males, after comparing these graphs with behaviors observed in the field and timed to an accuracy of 1 s. We used sequences of a sample of display runs identified and ground-truthed through field observations of some focal males during the main breeding season (2018–19) to train the model of the phyton-based *AcceleRater* application (Resheff et al. 2014), and so obtained display runs automatically for the 2019–20 breeding season, instead of having to get these sequences through visual inspection of all ACC-sequences of that year, as we did in 2018–19. Once all display runs were quantified and located on a map, we checked that the modal values for each male matched the 2018–19 display location. We selected display sites that were randomly distributed over the entire distribution area on Lanzarote and Fuerteventura (Figure 1). The distribution was plotted using ArcGIS Pro version 2.9 (ESRI 2021) as a combination of the kernel 95 and an aggregate of 1 km buffer zones around the coordinates of all houbaras sighted during the population censuses of both islands. Once the distribution area was obtained, 98 random points were generated within that area using the “*create random points*” tool of ArcGIS. For the generation of random points, an exclusion buffer of 100 m was applied around each location of a male houbara and 300 m if the male houbara was seen on display, to minimize the probability of overlapping random points with actual display sites (Morales et al. 2008; Alonso et al. 2012a).

### Variables used

**Figure 3:**
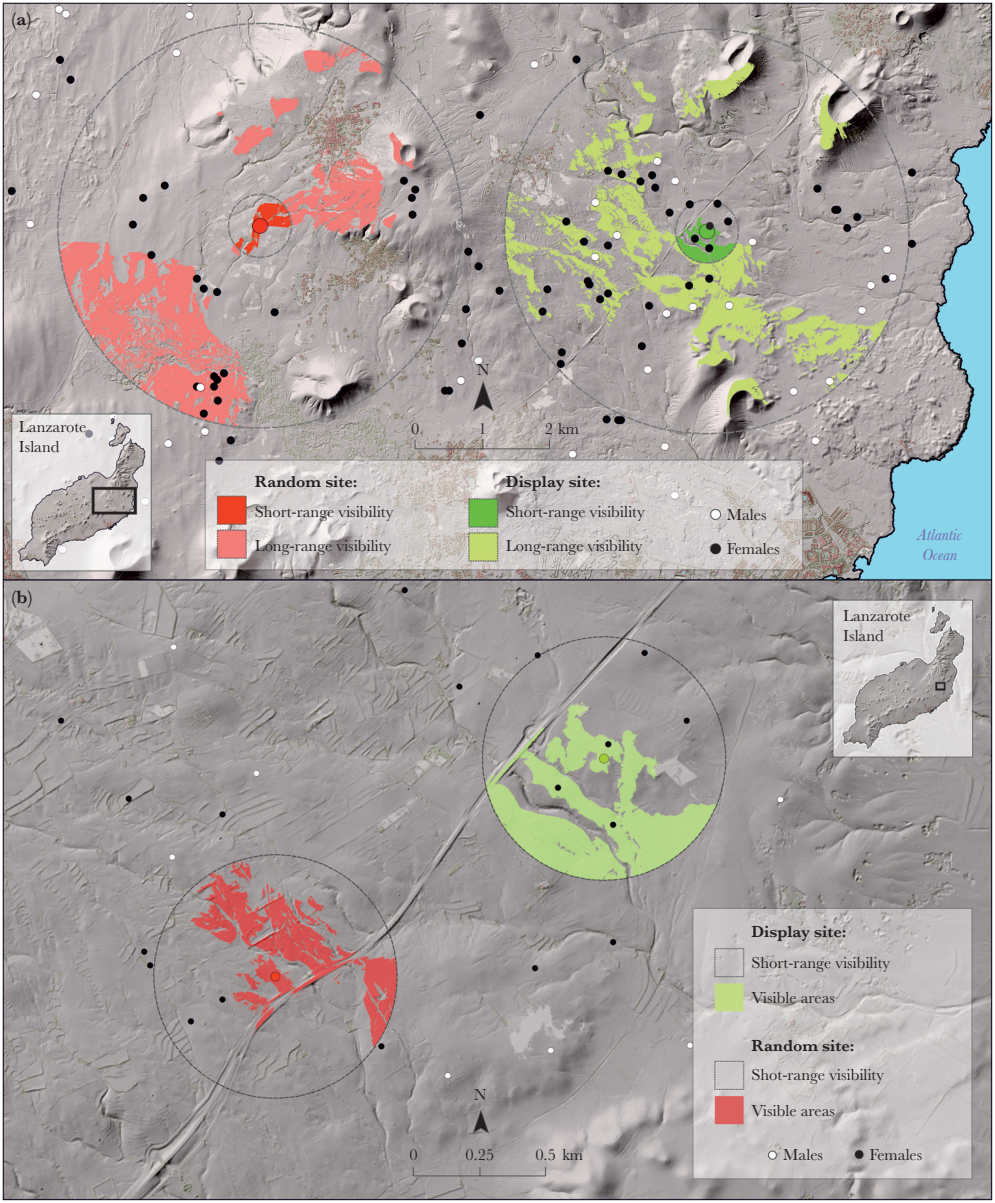
Maps of two sectors of the study area showing two Canarian houbara display sites and two random sites, overlaid on top of the DEM. The circles around display and random sites represent the two buffers (467 m and 3000 m, map A), or only the 467 m buffer (map B). Within the buffers, the area visible from the display site (green) and random site (red) are shown. Black dots are females and white dots are males, as recorded during the breeding population census. In example A, it can be appreciated that many more females and males are found within both buffers around the display site compared to the random site. Example B illustrates how a displaying male can see more females in the short-range buffer than it would see from a random site. The viewshed extends well off to the south, where at least 3 female locations exist.

To characterize display and random sites we used two scales: 1) the landscape scale, including environmental variables that affect selection on a broad scale (viewshed, female and male distribution, human infrastructure), and 2) the display site scale, to describe in detail the microhabitat structure of the site where the male performed display runs (Table 1, Figure 3):

**Table 1:** Differences (Mann–Whitney test) between display sites of male Canarian houbara bustards and random sites in relation to visibility, distribution of females and males, microhabitat structure and distance to human infrastructure

Variable (units)	Display site	Random site	*U*	*P*
	(Mean ± SD)	(Mean ± SD)		
Visibility				
Long-range visibility (up to 3000 m) (ha)	466.11 ± 236.03	240.49 ± 176.741	2026	<0.001
Short-range visibility (up to 467 m) (ha)	26.32 ± 11.72	14.93 ± 10.357	2170	<0.001
Female and male distribution				
Number of females in the 3000 m buffer	21.33 ± 16.67	18.17 ± 14.396	4312.5	0.217
Number of males in the 3000 m buffer	22.62 ± 13.65	23.39 ± 14.982	4259.5	0.641
Number of females in the 467 m buffer	0.95 ± 1.88	0.86 ± 1.910	4617	0.100
Number of males in the 467 m buffer	0.86 ± 1.03	0.76 ± 1.140	4341	0.204
Number of females visible in the 3000 m buffer	2.73 ± 2.76	1.15 ± 1.80	2996.5	<0.001
Number of males visible in the 3000 m buffer	5.77 ± 5.61	2.68 ± 3.81	2966	<0.001
Number of females visible in the 467m buffer	0.27 ± 0.73	0.12 ± 0.63	4190	0.004
Number of males visible in the 467 m buffer	0.49 ± 0.71	0.30 ± 0.60	4069	0.023
Microhabitat structure				
Central 1 × 1 m sample (C):				
Vegetation cover (%)	3.05 ± 4.75	26.91 ± 25.87	1695	<0.001
Dominant plant species height (cm)	1.15 ± 2.60	8.36 ± 13.86	2408	<0.001
Maximum vegetation height (cm)	1.53 ± 3.22	10.66 ± 13.55	1955.5	<0.001
Cover of small stones (2–10 cm) (%)	3.15 ± 7.06	5.73 ± 10.67	4129.5	0.067
Cover of large stones (>10 cm) (%)	1.49 ± 4.68	6.88 ± 15.91	3804.5	0.002
Roughness (cm)	3.48 ± 4.81	5.53 ± 6.60	3528	0.001
Biomass of arthropods (g)	3.01 ± 11.40	6.75 ± 38.92	4439.5	0.134
Biomass of mollusks (g)	1117.07 ± 4795.87	426.17 ± 2258.44	4498.5	0.179
Total biomass (g)	1120.09 ± 4795.43	432.92 ± 2258.34	4709.5	0.754
Mean of five 1 × 1 m samples (C, N, S, E, O)				
Biomass of arthropods (g)	1.98 ± 5.25	4.75 ± 15.65	4249.5	0.097
Biomass of mollusks (g)	411.96 ± 1485.55	489.45 ± 1679.65	4714	0.757
Total biomass (g)	413.53 ± 1485.43	492.02 ± 1679.38	4592.5	0.570
Human infrastructure				
Distance to main power lines (66 kV) (m)	8630.34 ± 3958.58	8011.12 ± 4124.20	4450	0.375
Distance to secondary power lines (15–20 kV) (m)	1470.55 ± 981.66	1394.07 ± 901.94	4733	0.862
Distance to road (m)	998.45± 675.42	865.10± 557.72	4390	0.299
Distance to track (m)	249.18 ± 174.20	197.71 ± 203.64	3624.5	0.003
Distance to building (m)	664.68 ± 373.70	562.16 ± 358.08	3959	0.034
Distance to urban nucleus (m)	1541.06 ± 701.99	1352.79 ± 772.20	4023	0.050

Sample size = 98 display/random sites. See Section 2 for a definition of variables.

#### Viewshed

We calculated the area visible from each display site as the number of pixels that could be seen from it, that is, with no other pixel blocking the visual line between the display site and the focal pixel, within a circular buffer around each display site. Analyses were carried out using a high-resolution digital elevation model (DEM) of 2 × 2 m pixel size obtained from LIDAR imaging at the Canary Islands Spatial Data Infrastructure (SDI) (https://www.idecanarias.es). This GIS analysis determines the raster surface locations visible to each observer feature, or in other words, identifies whether the observer point (i.e. the displaying male) is visible from each raster surface location (Law and Collins 2019). This made it possible to obtain very accurate viewsheds of the display site surroundings. The visibility of each cell center is determined by comparing the altitude angle to the cell center with the altitude angle to the local horizon. The local horizon is computed by considering the intervening terrain between the point of observation and the current cell center. If the point lies above the local horizon, it is considered visible. To account for the height of a male or a female observing or being observed, we added an offset height of 0.3 m to the display site and to all other pixels in the visibility analysis. We made all calculations for two different buffers, a long-range and a short-range visibility area, to account respectively for the maximum distance from which a displaying male would be seen by other houbaras (long-range), and for the visibility of the displaying male within its territory (short-range) (Figure 3). In the case of long-range buffer areas, we set the limit at 3000 m radius from each display site based on published visual acuity values of birds (Hodos 1993), and estimates from experts for other bustard species (G. Martin, personal communication; Alonso et al. 2012a). Field trials showed that a displaying male houbara bustard can be seen by the human eye at approximately 1–1.5 km (personal observation). Assuming visual acuity to be 2.5 times that of the human eye (Hodos 1993), a displaying male would certainly be visible at 3 km to a female (potential mate) or to another male (potential competitor). As for the short-range viewshed, we set the limit at 467 m, the mean radius of a circle of the same surface as that of an average male territory during the peak display season (own unpubl. data; based on 95% kernel density estimates of the areas used by our males in the breeding period).

#### Female and male distribution

We also examined where display and random sites were located in relation to the distribution of females and males. Specifically, we aimed to test whether display sites were located in areas with a higher presence of females and males than random sites and, more importantly, whether from display sites males could see (and were more visible to) more females and males than from random sites (Figure 3). To do this, we used female and male locations from surveys conducted in January and February 2018 on the island of Lanzarote and in January 2020 and 2021 on the island of Fuerteventura (for a detailed description of survey methods, see Alonso et al. 2020). We compared the distribution of females and males in relation to display site or random sites using 4 variables, two of them taking into account the individuals present in a buffer of 3000 m and two in a buffer of 467 m (Table 1 and Figure 3).

#### Microhabitat structure

A 1 × 1 m square was used to measure these variables (Martín et al. 1996; Mostacedo and Fredericksen 2000; Morales et al. 2008). First, the variables were measured at the center of the display site or random point (central sample, C), to define the microhabitat characteristics of the exact place where the male performs the circular display runs. Measurements at C were not taken exactly in the center of the display site (which is a circle of ca. 2 m diameter, see Figure 2), but directly on the track used by the male in its circular runs. Subsequently, the same variables were measured at four additional points by moving 10 m from C towards each of the four cardinal directions (N, S, E, W). The data from the five squares were averaged to quantify the microhabitat characteristics of the immediate surroundings of each display site or random point (Morales et al. 2008). We used eleven variables: 1) *vegetation cover*: percentage of the area of the 1 × 1 m square covered by vegetation; 2) *dominant plant species* and its *height*: we determined which was the dominant plant species inside the 1 × 1 m square and measured its height in cm; 3) *maximum height of vegetation*: we measured the maximum height of the vegetation found inside the 1 × 1 m square in cm; 4) *small stone cover (2–10 cm)*: percentage of the 1 × 1 m square covered by stones of a diameter between 2 and 10 cm; 5) *large stone cover (>10 cm)*: percentage of the area of the 1 × 1 m square covered by stones of a diameter larger than 10 cm; 6) *roughness*: vertical distance in cm from the highest point to the lowest point within the 1 × 1 m square (as a measure of the relief within the square; Rodrigues 1994); 7) *type of terrain*: qualitative classification of the type of substrate and vegetation composition (sandy plains with sparse vegetation -local name *jable*-, sandy plains with vegetation, cultivated field, abandoned cultivation, fallow field, lava field -local name, *malpaís*-, abandoned cultivated field covered with volcanic sand, stony field, stony field with vegetation, track); 8) *plant species composition*: plant species found in the 1 × 1 m square; 9) *biomass of arthropods:* total weight estimated for all arthropods found inside the 1 × 1 m square applying, for each arthropod order separately, the formula *W* = a*BL^*b* established by Hódar (1996); 10) *biomass of mollusk*: to estimate the total weight of mollusks in each 1 × 1 m square, we first calculated the average dry weight of a sample of 93 snails (= 632.97 mg), and multiplied it by the number of individuals found in each 1 × 1 m square; 11) *total biomass*: sum of the biomass of arthropods and mollusks in each 1 × 1 m square (no vertebrates were found in our samples).

#### Human infrastructure

Finally, to explore the possible effects of human infrastructure on display site selection, we calculated the Eucliden distance to six variables: *Distance to main power line*: straight-line distance from a display or random site to the nearest power line of 66 kV; *Distance to secondary power line*: straight-line distance from a display or random site to the nearest line of 15–20 kV (distribution line); *Distance to road*: straight-line distance from a display or random site to the nearest road (highway, main or secondary asphalt road); *Distance to track*: straight-line distance from a display or random site to the nearest track (unpaved road, main or secondary track); *Distance to nearest building*: straight-line distance from a display or random site to the nearest building (country house, farmhouse, tack room, etc); *Distance to the nearest urban center*: straight-line distance from a display or random site to the nearest city, town or urbanized area. The GIS database of human infrastructure (BTN25) was obtained at scale 1:200,000 from CNIG (2021) https://centrodedescargas.cnig.es/CentroDescargas/index.jsp.

### Statistical analyses

In a first step, we performed univariate comparisons between display and random points for all predictor variables defined using IBM SPSS Statistics 19 (IBM Company 2010). We used Mann–Whitney *U* tests, because data were generally not normally distributed (Kolmogorov–Smirnov and Shapiro–Wilk, *P* < 0.01). To analyze the dominant plant formation at display and random sites, we used the variable *dominant plant species* at C of each site. For this purpose, these species were grouped in two categories (shrubs or herbaceous), and a third category “bare ground” was considered for cases with no vegetation. Subsequently, we analyzed which was the dominant species in the area surrounding the display site or random point using the four additional samples (N, S, E, W). We excluded C because display sites usually have sparse or no vegetation due to male runs. Next, we analyzed the type of terrain selected by displaying male houbaras. For these three analyses (dominant plant formation, dominant species, type of terrain) a Chi-Square was used. Finally, we assessed the differences in plant species composition by quantifying the mean presence of flora species in the immediate surroundings of display and random sites, by means of Wilcoxon matched-pairs signed-rank tests. In these analyses of plant species composition, we also excluded C.

In a second step, we built Generalized Linear Models (GLM) with binomial errors and logit link function (McCullagh and Nelder 1989) to identify the most important among all possible predictors by using uncorrelated predictors and display location/random point as response variable. We used software R version 3.6.3 (https://www.r-project.org), packages “*rhr*” (Signer and Balkenhol 2015), “*MuMIn*” (Barton 2016) and “*lme4*” (Bates et al. 2015). Untransformed variables were used for GLM analyses, as normality is not required (Guisan and Zimmermann 2000). To reduce collinearity, we previously obtained a correlation matrix including all predictors and excluded the one with the lowest biological significance from all pairs of correlated variables (*r*_S_ > 0.7, Spearman correlation) (Randin et al. 2006). Six additional variables were deleted because they provided information very similar to other variables already included in the analyses. These were cover of small stones, mean of five samples of % vegetation cover, roughness, mean of five samples of roughness, biomass of arthropods and mean of five samples of biomass of arthropods (Supplementary Table S1). All possible subsets of predictor variables were analyzed and Akaike’s information criterion (AICc) was used to select the best subset. The values of ΔAICc and Akaike’s weight (ωAICc) were also calculated. Models with ΔAIC < 2 are considered to be substantially supported by the data and similar to the best model in their empirical support (Burnham and Anderson 2002; Heinze et al. 2018). With all candidate models, we performed an average model estimation, in which the parameter estimates of all models were combined by taking into account their corresponding Akaike weight (ωAICc) (Burnham and Anderson 2002). Finally, to estimate the relative importance of each variable, we calculated, for each predictor, the sum of Akaike weights of the models in which the predictor was present (∑), the mean and standard deviations of the regression coefficient (*b*), the *Z*-value (*Z*), the *P*-value (*P*) and the mean values of the 95% confidence interval for b (CI). GLMs were carried out using the function “*glmer*” of package “*lme4*” (Bates et al. 2015). There were no collinearity problems with variables.

### Ethical note

Capture, handling and marking houbara bustards were authorized and conducted under permissions issued by regional authorities (Viceconsejeria de Medio Ambiente, Gobierno de Canarias, license 2015/10584). The weight of the logger plus harness material was below the commonly accepted limit of 5% of the weight of the birds. We did not observe any stress signs or behavioral alteration of the birds from marking. The methods used comply with the Spanish guidelines for ethical use in animal research.

## RESULTS

The visibility from male display sites was significantly higher than from random points, both in the long-range and short-range (Table 1 and Figure 3). More interesting, although the numbers of males and females distributed in the long- and short-range buffers around the display site did not differ significantly from those present in the buffers around random points, males standing at their display sites could see (i.e. in their visible areas) more females and males within both buffers than they would see from random sites (125% increase in females, 63% in males, in the short-range buffer; 137% increase in females, 115% in males, in the long-range buffer; Table 1 and Figure 3). The analysis of microhabitat characteristics showed that display sites had lower vegetation cover and vegetation height than random sites (Table 1). In the central 1 × 1 m sample of display sites there was frequently bare soil, whereas in random sites the ground was more often covered with shrub or herbaceous vegetation (Chi-square = 40.30, *df* = 2, *P* < 0.001; Table 2). However, display sites often had a bush (most often *Launaea arborescens,* but also *Salsola vermiculata, Bassia tomentosa, Traganum moquinii, Lycium intricatum*) or a large herbaceous plant (most frecuently *Cenchrus ciliaris*) exactly in the center of the circular display track, and the track was often quite visible on the ground, reflecting the permanent use of the same circle by the male over the whole season (see Figure 2). There were no significant differences between display and random sites in the species composition of the plants in the immediate surroundings (four 1 × 1 m samples taken N, S, E and O of the central sample; Supplementary Table S2). Also, no significant differences were found in dominant species between display and random sites (Chi-square = 12.82, *df* = 12, *P* = 0.382), with the category “no dominant plant species” being the most frequent in both cases (50% at display sites, 32% at random sites). Finally, we found differences between display and random sites regarding the type of terrain (Chi-square = 33.15, *df* = 8, *P* = 0.006), with a majority of display sites being found in sandy habitat (*jable*) with vegetation (39.80%), while the most frequent terrain of random sites was abandoned crops (25.51%) (Chi-square = 33.15, *df* = 8, *P* = 0.006; Supplementary Table S3). As for the terrain structure, display sites had fewer large stones (diameter >10 cm) than random sites, and also showed fewer small stones, although the latter difference was only marginally significant (Table 1). Overall, the terrain roughness was significantly higher in random sites (Table 1). The variables related to arthropod and mollusk biomass showed no substantive differences between display and random sites (Table 1). Finally, display sites were found to be significantly farther away from urban nuclei, buildings and tracks than random locations (Table 1). No significant differences were found in the distances to power lines and roads.

**Table 2:** Percentages of the two major types of dominant plant formation (herbaceus, shrub) and bare soil found in the central 1 × 1 m sample of display and random sites

Dominant plant formation	% Display site	% Random site
Shrub vegetation	8.16	31.63
Herbaceous vegetation	29.59	48.98
Bare soil	62.25	19.39

The results of GLMs for display site selection using all independent predictors for visibility, distribution of females and males, microhabitat-related variables and distances to human infrastructure showed fifteen plausible candidate models (Table 3, Supplementary Table S4). The best of these models retained seven variables related to vegetation and rock cover, long- and short-range visibility, females visible within both buffers (467 and 3000 m), and males visible within the long-range buffer. The secondbest model, with almost the same weight and identical ΔAICc, did not include females visible at 467 m. The variables % vegetation cover, % rock cover >10 cm, short-range visibility and females visible at 3000 m were retained in all fifteen most plausible models (AICc ≤ 2). Furthermore, after model averaging, these four variables were found to be significant (Table 4 and Supplementary Table S5). Distance to urban nuclei appears in the third, fourth, eleventh and thirteenth models. Distance to tracks was also retained in models five, six, eleven and twelve (Table 3). None of these infrastructure variables were significant after model averaging (Table 4 and Supplementary Table S5). Finally, regarding visibility, it should be noted that models eight and ten did not retain the long-range visibility, a variable that was also discarded among the most influential ones during the process of model averaging (Table 4 and Supplementary Tables S5). The 16 marked males for which we had data in both years used the same display sites year after year.

**Table 3:** Candidate Generalized Linear Models (GLM) analyzing the effect of visibility, distribution of females and males, microhabitat, and distance to human infrastructure on display site selection in male Canarian houbara bustards

Model	AICc	*df*	ΔAICc	ωAICc
VEGCOV + ROCKCOV + VISIBSHORT + VISIBLONG + FEMVISIB3000 + MALEVISIB3000 + FEMVISIB467	123.2	8	0.00	0.13
VEGCOV + ROCKCOV + VISIBSHORT + VISIBLONG + FEMVISIB3000 + MALEVISIB3000	123.2	7	0.00	0.12
VEGCOV + ROCKCOV + VISIBSHORT + VISIBLONG + FEMVISIB3000 + MALEVISIB3000 + DISTURBAN	123.7	8	0.50	0.11
VEGCOV + ROCKCOV + VISIBSHORT + VISIBLONG + FEMVISIB3000 + MALEVISIB3000 + FEMVISIB467 + DISTURBAN	123.7	9	0.50	0.10
VEGCOV + ROCKCOV + VISIBSHORT + VISIBLONG + FEMVISIB3000 + MALEVISIB3000 + DISTTRACK	124.1	8	0.90	0.08
VEGCOV + ROCKCOV + VISIBSHORT + VISIBLONG + FEMVISIB3000 + MALEVISIB3000 + FEMVISIB467 + DISTTRACK	124.2	9	1	0.03
VEGCOV + ROCKCOV + VISIBSHORT + VISIBLONG + FEMVISIB3000	124.8	6	1.60	0.05
VEGCOV + ROCKCOV + VISIBSHORT + FEMVISIB3000 + MALEVISIB3000	124.8	6	1.60	0.05
VEGCOV + ROCKCOV + VISIBSHORT + VISIBLONG + FEMVISIB3000 + MALEVISIB467	124.9	7	1.70	0.05
VEGCOV + ROCKCOV + VISIBSHORT + FEMVISIB3000 + MALEVISIB3000 + FEMVISIB467	125	7	1.80	0.05
VEGCOV + ROCKCOV + VISIBSHORT + VISIBLONG + FEMVISIB3000 + MALEVISIB3000 + DISTURBAN + DISTTRACK	125	9	1.80	0.06
VEGCOV + ROCKCOV + VISIBSHORT + VISIBLONG + FEMVISIB3000 + MALEVISIB3000 + FEMVISIB467 + DISTURBAN + DISTTRACK	125	10	1.80	0.06
VEGCOV + ROCKCOV + VISIBSHORT + VISIBLONG + FEMVISIB3000 + MALEVISIB467 + DISTURBAN	125.1	8	1.90	0.05
VEGCOV + ROCKCOV + VISIBSHORT + VISIBLONG + FEMVISIB3000 + MALEVISIB3000 + MALEVISIB467	125.2	8	2	0.05
VEGCOV + ROCKCOV + VISIBSHORT + VISIBLONG + FEMVISIB3000 + MALEVISIB3000 + FEMVISIB467 + MALEVISIB467	125.2	9	2	0.03

Sample of 98 display/random sites identified for the Canarian houbara bustard. We show the best 15 models ranked from best to worst according to ΔAICc (AICc ≤ 2). Summary statistics include the corrected Akaike Information Criterion (AICc), df (degrees of freedom), difference in AICc (ΔAICc), and Akaike weight (ωAICc). The following 11 predictors were included in these GLMs: vegetation cover (VEGCOV), cover of large stones (diameter >10 cm) (ROCKCOV), long-range visibility (ha) (visibility up to 3000 m) (VISIBLONG), short-range visibility (ha) (visibility up to 467 m) (VISIBSHORT), number of females visible from the display/random site within a 3000 m-radius buffer (FEMVISIB3000), number of males visible from display/random site within a 3000 m-radius buffer (MALEVISIB3000), number of females visible from display/random site within a 467 m-radius buffer (FEMVISIB467), number of males visible from display/random site within a 467 m-radius buffer (MALEVISIB467), distance to nearest tracks or unpaved roads (DISTTRACK), distance to nearest building (DISTBUILD), distance to nearest urban nuclei (DISTURBAN) (see Section 2 for details).

**Table 4:** Model-averaged estimates of the display site predictor variables selected in the 15 most plausible models shown in Table 3

Predictor	∑	*b*	*SE*	*Z*	*P*	Lower CI	Upper CI
VEGCOV	1	−0.179	0.040	4.457	<0.001	−0.258	−0.100
ROCKCOV	1	−0.130	0.041	2.965	0.003	−0.205	−0.041
VISIBSHORT	1	0.071	0.026	2.642	0.008	0.018	0.122
FEMVISIB3000	1	0.532	0.169	3.086	0.002	0.191	0.855
VISIBLONG	0.90	0.002	0.002	1.387	0.121	−0.0002	0.005
MALEVISIB3000	0.90	0.090	0.066	1.354	0.172	−0.009	0.220
FEMVISIB467	0.40	0.471	0.761	0.653	0.530	−0.268	2.666
DISTURBAN	0.38	−0.0002	0.0003	0.0003	0.610	−0.119	0.0003
DISTTRACK	0.23	0.0002	0.001	0.366	0.733	−0.001	0.003
MALEVISIB467	0.18	0.074	0.236	0.271	0.768	−0.555	1.181

Values given indicate the relative importance (∑, sum of Akaike weights of the models in which the predictor was present), regression coefficients (*b*), standard errors (*SE*), *P*-values, *Z*-values (*Z*) and 95% coefficient intervals for *b* (CI). See definitions of variables in Table 3.

## DISCUSSION

Our results show that male Canarian houbara bustards select display sites with low vegetation and stone cover and a large viewshed, located in places that tend to be away from urban nuclei, buildings and tracks. These characteristics allow them to perform display runs in terrain free of obstacles such as rocks and plants, minimize human disturbance and predation risk, and maximize the transmission of sexual signals to surrounding females and males. This study represents the first detailed analysis of display site selection in the *Canarian* subspecies of African houbara bustard. Our study included what we thought to be the most important predictor variables, and the main contributions compared to previous studies were the viewshed analyses, and the fact that we were able to relate visibility to the distribution of houbara bustards at the time of the study. We obtained very accurate viewsheds of the surroundings of 98 displaying males using a high-resolution digital elevation model created from LIDAR technology (Neumann et al. 2015; Sharma et al. 2021), which has become available only recently and represents a breakthrough in viewshed ecology (Aben et al. 2018; Sahraoui et al. 2018; Zong et al. 2021). Although some authors had already mentioned the importance of visibility, no previous study had quantified the viewshed or related to the actual distribution of the species. For example, Hingrat et al. (2008) used a conspicuousness index estimated with a naked eye from the visibility to human observers of a dummy placed on the ground, Geary et al. (2022) used a very small sample size of only ten displaying males and did not include visibility as a predictor, and other authors studied display site selection using only vegetation or substrate variables but did not include visibility (Yang 2002; Koshkin et al. 2016).

Among microhabitat variables that were significant in the model averaging assessment, vegetation and stone cover were the most important. This result was to be expected, since a terrain free of plants and rocks is necessary for the male to be able to run without having to look at the ground to prevent injuring its tarsi, especially considering that, while running, its head is partially hidden by the display plumage. This explains why most display sites were on sandy ground, and why the sandy areas of the islands known as *jable* show the highest breeding densities. The selection of display sites with low vegetation cover and height allows males to be more visible to conspecifics (sexual advertisement hypothesis), minimizing obstacles that could block visual and also vocal signal transmission (Cornec 2015; Cornec et al. 2017), as well as facilitating the detection of potential predators (predator avoidance hypothesis). Similar results were found in previous studies (Yang 2002; Cuziat et al. 2005; Hingrat et al. 2008; Koshkin et al. 2016). The constant trampling by the male during circular runs favors the maintenance of bare ground, even between years, and explains why most display sites were non-vegetated ground, compared to the more grassy or shrubby random sites. An interesting observation in our study was the frequent presence of a shrub in the center of the display site (Figure 2). A probable explanation for the males’ preference to choose these shrubs as the center of their circular display runs is that they can quickly stop displaying and hide behind the shrub, either standing or laying down, when they perceive a risk (pers. obs.) (Supplementary Figure S1).

Display sites were not characterized by a high invertebrate biomass. Indeed, they showed a marginally significant trend to have a lower arthropod biomass compared to random sites, which may be due to males prioritizing sites with good visibility and thus with sparser vegetation, over sites with more abundant resources that they could use themselves or for attracting females. On the other hand, no significant differences were found in plant species composition, dominant plant formation and dominant plant species. In our study area, as well as in Morocco where the nominate subspecies is found, food resources are abundant and evenly distributed (Hingrat et al. 2007; Abril-Colón et al. 2022b), so they were not economically defensible for males (Hingrat and Saint Jalme 2005). Emlen and Oring (1977) argued that, when resources are defensible, territoriality was generally promoted and males tend to distribute regularly. The mating system of houbara bustards has been found to fit the definition of an exploded lek, with males displaying at distances of several hundred meters from each other, defending their display sites and small territories around them against other males (Collins 1984; Hingrat et al. 2004, 2008; Hingrat and Saint Jalme 2005; Alonso et al. 2020). Despite having found that the amount of food resources was not higher at display sites, male territories generally included some vegetation where males could feed, rest or hide (Martín et al. 1996; Launay et al. 1997; Osborne et al. 1997). However, we observed that many males defended their display site from intruders, but often gathered with neighbours at midday for feeding. Therefore, Canarian houbaras seem to follow a display-site defense model, where display sites contain no resources valuable for females (Bradbury 1981), although we cannot completely discard resource defense or territorial polygyny occurring for some individuals, as happens in other bustard species (see e.g. Alonso et al. 2012b). On the other hand, males spend most of their time displaying and only a limited time feeding or resting. Thus, given that display behavior is energetically demanding (Vehrencamp et al. 1989), the presence of sufficient food was probably important when deciding where to establish the territory. Other authors have found that easy access to food resources seems to be important in display site selection (Yang 2002; Hingrat et al. 2007).

Certainly, the most interesting result of our study was the identification of visibility as a key factor in display site selection. Both, long- and short-range visibilities were retained in the two most plausible GLMs. But more importantly, we found that from display sites males could see more females and males, both at long and short distances, in spite of the total number of nearby males and females not being statistically different between display and random sites. This may explain why males use the same display sites year after year and clearly indicates that males have a very accurate image of the topography of their territory, but also of the distribution of conspecifics, both within and beyond the territory limits. Although the numbers of males and females were based on just a single population survey carried out in January, and undoubtedly the birds moved around these locations, the fidelity of both sexes to their locations and the limited amplitude of their movements during the mating season (Abril-Colón et al. 2022a) suggest that the January survey locations were reasonable proxies of their average positions to check the hypotheses regarding their presence within males’ viewsheds.

This result shows that it is important not only to use a display site with good visibility, but to use one from which the male can see, and be seen by a high number of females and males. In other words, among all possible high points with good viewshed, males selected those that were located in the best possible sites to see both potential mates and competing males. Model averaging helped us identify short-range visibility and females visible within a 3000 m-radius buffer as the two most important of the variables that predicted display site seleccion in males. A reasonable interpretation is that displaying males tried to maximize the viewshed over their territories (short-range buffer) but at the same time also the number of females in view at any distance. These results provide further, and more refined support for the hypotheses of sexual advertisement and predator avoidance than a simple viewshed difference between display and random points. Maximization of numbers of females and males visible within the long-range buffer highlights the importance of transmitting sexual signals over long distances, which is an essential feature of the exploded lek, where males defend relatively small territories but transmit sexual signals to reach as many females and males as possible. The display of male houbaras, like that of many lekking birds, has clearly evolved to transmit visual and acoustic signals at large distances, and not just within the boundaries of a territory (Johnsgard 1994; Höglund and Alatalo 1995; Cornec et al. 2017).

Finally, regarding human disturbance sources, univariate analyses showed that display sites were farther away from urban nuclei, buildings and tracks than random points. In our study area, several major tracks cross important houbara breeding areas, and are heavily used by vehicles. Surprisingly, Hingrat et al. (2008) noted that, in Morocco, display sites were closer to tracks than random sites, but this may be explained by the much lower traffic intensity in the Moroccan desert compared to our study area, a top tourist destination where tracks are intensively used by hikers, cyclists, cars and off-road vehicles (Carrascal et al. 2006, 2008; Banos-González et al. 2016). In Morocco, some males even displayed directly in the middle of tracks, probably because tracks provided artificial open places without stones and vegetation and thus suitable for running display. As for urban nuclei, broad habitat selection studies have also found that houbara presence decreases with proximity to human settlements (Cuziat et al. 2005; Carrascal et al. 2006, 2008; Hingrat et al. 2008; Chammem et al. 2012; Schuster et al. 2012). Regarding roads and power lines, the fact that differences between display and random sites did not reach statistical significance in univariate analyses and did not appear in the models, may have been due to our very restrictive criterial way to select random sites, within the area used by the species. We did so because we were interested in investigating the selection of display sites at a detailed scale, rather than at the general habitat selection scale of previous studies that found the distance to the nearest road, and also road density to be significant predictors of the distribution of houbara bustards in the Canary Islands (Carrascal et al. 2006, 2008; Schuster et al. 2012).

In sum, the results presented here highlight the importance of viewshed in display site selection, something that has been neglected in previous works. Among numerous variables analyzed, two related to visibility and visible conspecifics proved to be among the most important. Future studies should consider whether other species, particularly terrestrial birds living in open habitats and displaying on the ground, use similar mechanisms to optimize visual or acoustic signal transmission during sexual display.

### Conservation implications

To ensure adequate protection of display sites, the construction of new tracks should be avoided, and vehicle traffic restrictions should be considered on certain tracks during the breeding season. Furthermore, the proliferation of dispersed buildings in areas occupied by houbaras should be prohibited, and urban sprawl should be controlled. This would minimize disturbance to males during the mating season. With regard to vegetation, the current gorse *Launaea arborescens* plant cover should be maintained in the main breeding areas of the houbara bustard, and some areas should be set aside for the exclusive use of bustards, where goat grazing should not be allowed.

## Supplementary Material

arac112_suppl_Supplementary_Figure

arac112_suppl_Supplementary_Tables

## Notes

The authors would like to thank A. Rodríguez and all volunteers from Lanzarote and Fuerteventura for their collaboration during the fieldwork, E. Rico, A. Reyes and J. Cazorla for their help in determining the plants and P. Oromi for his help in determining the arthropods, S. Taheri for help with R software, M. Cabrera and A. Delgado for managing capture permits, and the guards of Cabildo of Lanzarote, Cabildo of Fuerteventura, and the Government of the Canary Islands for their collaboration. We thank the authorities of the Cabildo of Lanzarote, Cabildo of Fuerteventura, Organismo Autónomo de Parques Nacionales of La Graciosa, and the Consejería de Transición Ecológica, Lucha Contra el Cambio Climático y Planificación Territorial of the Canary Islands for the permits to work with houbara bustards.
