# Supplementary figures and images for: Display site selection in a ground dwelling bird: the importance of viewshed
Source: Behav Ecol. 2022 Dec 23;34(2):223–35. doi: 10.1093/beheco/arac112 (PMC10047634; doi:10.1093/beheco/arac112)

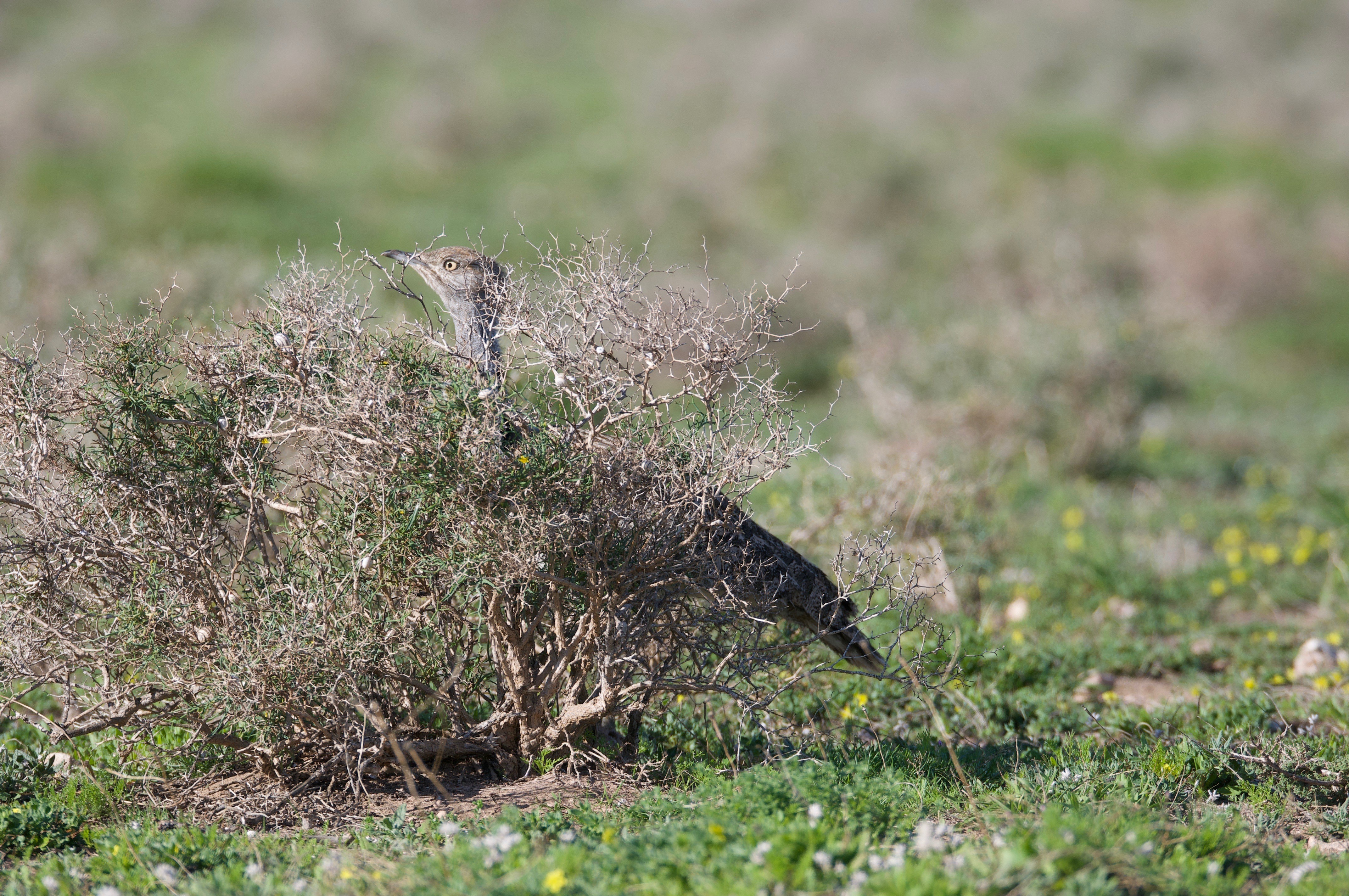

Supplement: arac112_suppl_Supplementary_Figure [file arac112_suppl_supplementary_figure.jpeg]
