# Supplementary material for: Display site selection in a ground dwelling bird: the importance of viewshed
Source: Behav Ecol. 2022 Dec 23;34(2):223–35. doi: 10.1093/beheco/arac112 (PMC10047634; doi:10.1093/beheco/arac112)
Supplement: arac112_suppl_Supplementary_Tables [file arac112_suppl_supplementary_tables.doc]

**Supplementary Table S1. List of all variables and the reasons why they were included/excluded in the generalized linear models (GLM).**

| Variable | Excluded or Included | Explanation |
| --- | --- | --- |
| *Visibility* | | |
| Long-range visibility (up to 3000 m) (ha) | Included | The long-range visible area is an important factor in the selection of the display site. |
| Short-range visibility (up to 467 m) (ha) | Included | The area visible at close range is an important factor in the selection of the display site. |
| Female and male distribution | | |
| Number of females in the 3000 m buffer | Excluded | This variable shows no significant differences between display and random sites in the univariate analysis (Table 1). |
| Number of males in the 3000 m buffer | Excluded | Same as above. |
| Number of females in the 467 m buffer | Excluded | Same as above. |
| Number of males in the 467 m buffer | Excluded | Same as above. |
| Number of females visible in the 3000 m buffer | Included | This is an important variable in the selection of the display site (sexual advertisement hypothesis). |
| Number of males visible in the 3000 m buffer | Included | Same as above. |
| Number of females visible in the 467m buffer | Included | Same as above |
| Number of males visible in the 467m buffer | Included | Same as above. |
| *Microhabitat structure* | | |
| *Central 1 x 1 m sample (C):* | | |
| Vegetation cover (%) | Included | The selection of display sites with low vegetation cover allows males to be more visible to conspecifics (sexual advertisement hypothesis), minimising obstacles that could block visual and also vocal signal transmission (Cornec 2015; Cornec et al. 2017), as well as facilitating the detection of potential predators (predator avoidance hypothesis). |
| Dominant plant species height (cm) | Excluded | This variable is correlated with vegetation cover (%). |
| Maximun vegetation height (cm) | Excluded | This variable is correlated with vegetation cover (%). |
| Cover of small stones (2-10 cm) (%) | Excluded | This variable is correlated with Cover of large stones (>10 cm) (%). |
| Cover of large stones (>10 cm) (%) | Included | A rock-free terrain is necessary for the male to be able to run without having to look at the ground to avoid the risk of injuring his tarsi, especially as his head is partially hidden by the display plumage while running. |
| Roughness (cm) | Excluded | This variable is correlated with cover of large stones (>10 cm) (%). |
| Biomass of arthropods (g) | Excluded | This variable shows no significant differences between display and random sites in the univariate analysis (Table 1). |
| Biomass of molluscs (g) | Excluded | Same as above. |
| Total biomass (g) | Excluded | Same as above. |
| *Mean of five 1 x 1 m samples (C, N, S, E, O)* | | |
| Biomass of arthropods (g) | Excluded | This variable shows no significant differences between display and random sites in the univariate analysis (Table 1). |
| Biomass of molluscs (g) | Excluded | Same as above. |
| Total biomass (g) | Excluded | Same as above. |
| *Human infrastructure* | | |
| Distance to main power lines (66 kV) (m) | Excluded | This variable shows no significant differences between display and random sites in the univariate analysis (Table 1). |
| Distance to secondary power lines (15-20 kV) (m) | Excluded | Same as above. |
| Distance to road (m) | Excluded | Same as above. |
| Distance to track (m) | Included | In the study area, trails are heavily used by hikers, cyclists, cars and off-road vehicles (Carrascal et al. 2006, 2008; Banos-González et al. 2016), causing much disturbance to houbaras. |
| Distance to building (m) | Included | Habitat selection studies have also found that houbara presence decreases with proximity to human settlements (Le Cuziat et al. 2005; Carrascal et al. 2006, 2008; Hingrat et al. 2008; Chammem et al. 2012; Schuster et al. 2012). |
| Distance to urban nucleus (m) | Included | Same as above. |

**Supplementary Table S2**. Comparison of plant species occurrence between display and random sites (Wilcoxon signed-rank test).

| **Dominant species** | **Display site presence (mean value)** | **Random site presence (mean value)** | **Z** | **p** |
| --- | --- | --- | --- | --- |
| *Launaea arborescens* (Batt.) Murb. | 0.37 | 0.41 | - 0.329 | 0.742 |
| *Lycium intricatum* Boiss. | 0 | 0.03 | - 1.000 | 0.317 |
| *Frankenia capitata* Webb & Berthel. | 0.13 | 0.14 | 0.000 | 1.000 |
| *Euphorbia balsamifera* Ait. | 0 | 0.05 | -1.342 | 0.180 |
| *Kleinia neriifolia* Haw. | 0 | 0.03 | - 1.000 | 0.317 |
| *Opuntia maxima* Mill. | 0 | 0.08 | - 1.000 | 0.317 |
| *Ficus carica* L. | 0 | 0.03 | - 1.000 | 0.317 |
| *Atriplex glauca* L. | 0 | 0.05 | - 1.000 | 0.317 |
| *Traganum moquinii* Webb ex Moq. in DC. | 0.13 | 0.27 | - 1.354 | 0.176 |
| *Chenoleoides tomentosa* (Lowe) Botsch. | 0.21 | 0.32 | - 0.780 | 0.435 |
| *Polycarpaea nivea* (Aiton) Webb | 0.26 | 0.41 | - 0.537 | 0.591 |
| *Suaeda vermiculata* Forssk. ex J. F. Gmel. | 0.11 | 0.11 | - 0.137 | 0.891 |
| *Helianthemum canariense* (Jacq.) Pers. | 0.03 | 0.11 | - 0.816 | 0.414 |
| *Androcymbium psammophilum* Svent. | 0.03 | 0.06 | - 0.447 | 0.655 |
| *Suaeda fruticosa* (L.) Forsk | 0.18 | 0 | -1.890 | 0.059 |
| Anuales verdes (*Lobularia*, *Notoceras*, *Calendula*) | 0.53 | 0.24 | - 1.281 | 0.200 |
| Dried herbaceous | 0.55 | 0.32 | - 0.984 | 0.325 |
| Herbaceous perennial | 0.34 | 0.57 | - 0.329 | 0.742 |
| Without plant species | 1.58 | 1.43 | - 0.327 | 0.744 |

The figures are mean occurrence values (presence = 1, absence = 0) of the four 1 x 1 m samples taken in the immediate surroundings (10 m north, south, east and west) of the display/random site center.

**Supplementary Table S3.** Distribution of the 98 display and random sites among the nine types of terrain identified in the study area.

| **Type of terrain** | **% Display sites**  **(n = 98)** | **% Random sites**  **(n = 98)** |
| --- | --- | --- |
| Sandy plains with sparse vegetation (*jable*) | 13.27 | 20.41 |
| Sandy plains with vegetation | 39.80 | 24.49 |
| Abandoned agricultural field | 24.49 | 25.51 |
| Lava fields (*malpaís*) | 6.12 | 8.16 |
| Abandoned agricultural field covered with volcanic sand | 4.08 | 6.12 |
| Stony field | 5.10 | 8.16 |
| Stony field with vegetation | 5.10 | 1.02 |
| Fallow field | 2.04 | 4.08 |
| Track | 0 | 2.04 |

**Supplementary Table S4**. Generalized Linear Models (GLM) explaining display site selection in male Canarian houbara bustards. Model averaging showing parameter estimates, confidence intervals (CI), adjusted error, Z-value (Z) and significance of all models. See definitions of variables in Table 3.

| **Covariate** | **Model** | **Parameter estimate** | **Lower CI** | **Upper CI** | **Adjusted SE** | **Z** | **p** |
| --- | --- | --- | --- | --- | --- | --- | --- |
| INTERCEPT | VEGCOV + ROCKCOV + VISIBSHORT + VISIBLONG + FEMVISIB3000 + MALEVISIB3000 + FEMVISIB467 | -1.729 | -3.112 | -0.495 | 0.661 | -2.614 | 0.009 |
|  | VEGCOV + ROCKCOV + VISIBSHORT + VISIBLONG + FEMVISIB3000 + MALEVISIB3000 | -1.695 | -3.068 | -0.468 | 0.657 | -2.58 | 0.009 |
|  | VEGCOV + ROCKCOV + VISIBSHORT + VISIBLONG + FEMVISIB3000 + MALEVISIB3000 + DISTURBAN | -1.257 | -2.774 | 0.159 | 0.741 | -1.696 | 0.089 |
|  | VEGCOV + ROCKCOV + VISIBSHORT + VISIBLONG + FEMVISIB3000 + MALEVISIB3000 + FEMVISIB467 + DISTURBAN | -1.275 | -2.808 | 0.156 | 0.748 | -1.703 | 0.088 |
|  | VEGCOV + ROCKCOV + VISIBSHORT + VISIBLONG + FEMVISIB3000 + MALEVISIB3000 + DISTTRACK | -2.083 | -3.688 | -0.663 | 0.764 | -2.725 | 0.006 |
|  | VEGCOV + ROCKCOV + VISIBSHORT + VISIBLONG + FEMVISIB3000 + MALEVISIB3000 + FEMVISIB467 + DISTTRACK | -1.704 | -3.187 | -0.378 | 0.709 | -2.404 | 0.016 |
|  | VEGCOV + ROCKCOV + VISIBSHORT + VISIBLONG + FEMVISIB3000 | -1.306 | -2.569 | -0.163 | 0.607 | -2.150 | 0.032 |
|  | VEGCOV + ROCKCOV + VISIBSHORT + FEMVISIB3000 + MALEVISIB3000 | -1.301 | -2.531 | -0.173 | 0.595 | -2.184 | 0.028 |
|  | VEGCOV + ROCKCOV + VISIBSHORT + VISIBLONG + FEMVISIB3000 + MALEVISIB467 | -1.490 | -2.793 | -0.314 | 0.626 | -2.380 | 0.017 |
|  | VEGCOV + ROCKCOV + VISIBSHORT + FEMVISIB3000 + MALEVISIB3000 + FEMVISIB467 | -1.337 | -2.584 | -0.199 | 0.602 | -2.219 | 0.026 |
|  | VEGCOV + ROCKCOV + VISIBSHORT + VISIBLONG + FEMVISIB3000 + MALEVISIB3000 + DISTURBAN + DISTTRACK | -1.638 | -3.401 | -0.010 | 0.857 | -1.911 | 0.055 |
|  | VEGCOV + ROCKCOV + VISIBSHORT + VISIBLONG + FEMVISIB3000 + MALEVISIB3000 + FEMVISIB467 + DISTURBAN + DISTTRACK | -1.665 | -3.451 | -0.015 | 0.868 | -1.918 | 0.055 |
|  | VEGCOV + ROCKCOV + VISIBSHORT + VISIBLONG + FEMVISIB3000 + MALEVISIB467 + DISTURBAN | -1.032 | -2.475 | 0.327 | 0.707 | -1.459 | 0.144 |
|  | VEGCOV + ROCKCOV + VISIBSHORT + VISIBLONG + FEMVISIB3000 + MALEVISIB3000 + MALEVISIB467 | -1.705 | -3.076 | -0.478 | 0.656 | -2.597 | 0.009 |
|  | VEGCOV + ROCKCOV + VISIBSHORT + VISIBLONG + FEMVISIB3000 + MALEVISIB3000 + FEMVISIB467 + MALEVISIB467 | -1.740 | -3.122 | -0.506 | 0.661 | -2.632 | 0.008 |
| VEGCOV | VEGCOV + ROCKCOV + VISIBSHORT + VISIBLONG + FEMVISIB3000 + MALEVISIB3000 + FEMVISIB467 | -0.181 | -0.270 | -0.115 | 0.039 | -4.608 | <0.001 |
|  | VEGCOV + ROCKCOV + VISIBSHORT + VISIBLONG + FEMVISIB3000 + MALEVISIB3000 | -0.177 | -0.270 | -0.111 | 0.040 | -4.420 | <0.001 |
|  | VEGCOV + ROCKCOV + VISIBSHORT + VISIBLONG + FEMVISIB3000 + MALEVISIB3000 + DISTURBAN | -0.176 | -0.269 | -0.110 | 0.040 | -4.393 | <0.001 |
|  | VEGCOV + ROCKCOV + VISIBSHORT + VISIBLONG + FEMVISIB3000 + MALEVISIB3000 + FEMVISIB467 + DISTURBAN | -0.180 | -0.269 | 0.114 | 0.039 | -4.579 | <0.001 |
|  | VEGCOV + ROCKCOV + VISIBSHORT + VISIBLONG + FEMVISIB3000 + MALEVISIB3000 + DISTTRACK | -0.174 | -2.655 | -0.108 | 0.039 | -4.389 | <0.001 |
|  | VEGCOV + ROCKCOV + VISIBSHORT + VISIBLONG + FEMVISIB3000 + MALEVISIB3000 + FEMVISIB467 + DISTTRACK | -0.184 | -0.275 | -0.118 | 0.039 | -4.641 | <0.001 |
|  | VEGCOV + ROCKCOV + VISIBSHORT + VISIBLONG + FEMVISIB3000 | -0.173 | -0.260 | -0.110 | 0.0378 | -4.584 | <0.001 |
|  | VEGCOV + ROCKCOV + VISIBSHORT + FEMVISIB3000 + MALEVISIB3000 | -0.185 | -0.278 | -0.116 | 0.041 | -4.519 | <0.001 |
|  | VEGCOV + ROCKCOV + VISIBSHORT + VISIBLONG + FEMVISIB3000 + MALEVISIB467 | -0.177 | -0.269 | -0.111 | 0.039 | -4.440 | <0.001 |
|  | VEGCOV + ROCKCOV + VISIBSHORT + FEMVISIB3000 + MALEVISIB3000 + FEMVISIB467 | -0.189 | -0.280 | -0.121 | 0.040 | -4.694 | <0.001 |
|  | VEGCOV + ROCKCOV + VISIBSHORT + VISIBLONG + FEMVISIB3000 + MALEVISIB3000 + DISTURBAN + DISTTRACK | -0.173 | -0.264 | -0.107 | 0.039 | -4.348 | <0.001 |
|  | VEGCOV + ROCKCOV + VISIBSHORT + VISIBLONG + FEMVISIB3000 + MALEVISIB3000 + FEMVISIB467 + DISTURBAN + DISTTRACK | -0.176 | -0.265 | -0.110 | 0.039 | -4.515 | <0.001 |
|  | VEGCOV + ROCKCOV + VISIBSHORT + VISIBLONG + FEMVISIB3000 + MALEVISIB467 + DISTURBAN | -0.177 | -0.270 | -0.111 | 0.041 | -4.375 | <0.001 |
|  | VEGCOV + ROCKCOV + VISIBSHORT + VISIBLONG + FEMVISIB3000 + MALEVISIB3000 + MALEVISIB467 | -0.178 | -0.273 | -0.111 | 0.041 | -4.378 | <0.001 |
|  | FEMVISIB3000 + MALEVISIB3000 + FEMVISIB467 + MALEVISIB467 | -0.182 | -0.274 | -0.115 | 0.040 | -4.566 | <0.001 |
| ROCKCOV | VEGCOV + ROCKCOV + VISIBSHORT + VISIBLONG + FEMVISIB3000 + MALEVISIB3000 + FEMVISIB467 | -0.117 | -0.204 | -0.042 | 0.040 | 2.899 | 0.004 |
|  | VEGCOV + ROCKCOV + VISIBSHORT + VISIBLONG + FEMVISIB3000 + MALEVISIB3000 | -0.122 | -0.210 | -0.047 | 0.040 | -3.012 | 0.003 |
|  | VEGCOV + ROCKCOV + VISIBSHORT + VISIBLONG + FEMVISIB3000 + MALEVISIB3000 + DISTURBAN | -0.140 | -0.239 | -0.060 | 0.044 | -3.146 | 0.002 |
|  | VEGCOV + ROCKCOV + VISIBSHORT + VISIBLONG + FEMVISIB3000 + MALEVISIB3000 + FEMVISIB467 + DISTURBAN | -0.135 | -0.233 | -0.055 | 0.044 | -3.056 | 0.002 |
|  | VEGCOV + ROCKCOV + VISIBSHORT + VISIBLONG + FEMVISIB3000 + MALEVISIB3000 + DISTTRACK | -0.124 | -0.215 | -0.051 | 0.040 | 3.047 | 0.002 |
|  | VEGCOV + ROCKCOV + VISIBSHORT + VISIBLONG + FEMVISIB3000 + MALEVISIB3000 + FEMVISIB467 + DISTTRACK | -0.129 | -0.216 | -0.056 | 0.040 | -3.247 | 0.001 |
|  | VEGCOV + ROCKCOV + VISIBSHORT + VISIBLONG + FEMVISIB3000 | -0.119 | -0.211 | -0.046 | 0.041 | -2.893 | 0.003 |
|  | VEGCOV + ROCKCOV + VISIBSHORT + FEMVISIB3000 + MALEVISIB3000 | -0.131 | -0.218 | -0.057 | 0.040 | -3.278 | 0.001 |
|  | VEGCOV + ROCKCOV + VISIBSHORT + VISIBLONG + FEMVISIB3000 + MALEVISIB467 | -0.134 | -0.227 | -0.055 | 0.042 | -3.125 | 0.002 |
|  | VEGCOV + ROCKCOV + VISIBSHORT + FEMVISIB3000 + MALEVISIB3000 + FEMVISIB467 | -0.127 | -0.213 | -0.053 | 0.039 | -3.198 | 0.001 |
|  | VEGCOV + ROCKCOV + VISIBSHORT + VISIBLONG + FEMVISIB3000 + MALEVISIB3000 + DISTURBAN + DISTTRACK | -0.141 | -0.242 | -0.060 | 0.045 | -3.132 | 0.002 |
|  | VEGCOV + ROCKCOV + VISIBSHORT + VISIBLONG + FEMVISIB3000 + MALEVISIB3000 + FEMVISIB467 + DISTURBAN + DISTTRACK | -0.137 | -0.237 | -0.056 | 0.045 | -3.045 | 0.002 |
|  | VEGCOV + ROCKCOV + VISIBSHORT + VISIBLONG + FEMVISIB3000 + MALEVISIB467 + DISTURBAN | -0.158 | -0.264 | -0.071 | 0.048 | -3.256 | 0.001 |
|  | VEGCOV + ROCKCOV + VISIBSHORT + VISIBLONG + FEMVISIB3000 + MALEVISIB3000 + MALEVISIB467 | -0.127 | -0.220 | -0.049 | 0.043 | -2.971 | 0.003 |
|  | VEGCOV + ROCKCOV + VISIBSHORT + VISIBLONG + FEMVISIB3000 + MALEVISIB3000 + FEMVISIB467 + MALEVISIB467 | -0.123 | -0.215 | -0.044 | 0.042 | -2.855 | 0.004 |
| VISIBSHORT | VEGCOV + ROCKCOV + VISIBSHORT + VISIBLONG + FEMVISIB3000 + MALEVISIB3000 + FEMVISIB467 | 0.066 | 0.016 | 0.119 | 0.026 | 2.531 | 0.011 |
|  | VEGCOV + ROCKCOV + VISIBSHORT + VISIBLONG + FEMVISIB3000 + MALEVISIB3000 | 0.067 | 0.019 | 0.119 | 0.025 | 2.654 | 0.007 |
|  | VEGCOV + ROCKCOV + VISIBSHORT + VISIBLONG + FEMVISIB3000 + MALEVISIB3000 + DISTURBAN | 0.071 | 0.021 | 0.125 | 0.026 | 2.701 | 0.007 |
|  | VEGCOV + ROCKCOV + VISIBSHORT + VISIBLONG + FEMVISIB3000 + MALEVISIB3000 + FEMVISIB467 + DISTURBAN | 0.069 | 0.0183 | 0.124 | 0.027 | 2.584 | 0.010 |
|  | VEGCOV + ROCKCOV + VISIBSHORT + VISIBLONG + FEMVISIB3000 + MALEVISIB3000 + DISTTRACK | 0.071 | 0.022 | 0.126 | 0.026 | 2.752 | 0.006 |
|  | VEGCOV + ROCKCOV + VISIBSHORT + VISIBLONG + FEMVISIB3000 + MALEVISIB3000 + FEMVISIB467 + DISTTRACK | 0.091 | 0.047 | 0.143 | 0.024 | 3.799 | <0.001 |
|  | VEGCOV + ROCKCOV + VISIBSHORT + VISIBLONG + FEMVISIB3000 | 0.064 | 0.017 | 0.116 | 0.024 | 2.594 | 0.001 |
|  | VEGCOV + ROCKCOV + VISIBSHORT + FEMVISIB3000 + MALEVISIB3000 | 0.087 | 0.044 | 0.136 | 0.023 | 3.787 | <0.001 |
|  | VEGCOV + ROCKCOV + VISIBSHORT + VISIBLONG + FEMVISIB3000 + MALEVISIB467 | 0.065 | 0.017 | 0.116 | 0.025 | 2.595 | 0.010 |
|  | VEGCOV + ROCKCOV + VISIBSHORT + FEMVISIB3000 + MALEVISIB3000 + FEMVISIB467 | 0.087 | 0.044 | 0.136 | 0.023 | 3.745 | <0.001 |
|  | VEGCOV + ROCKCOV + VISIBSHORT + VISIBLONG + FEMVISIB3000 + MALEVISIB3000 + DISTURBAN + DISTTRACK | 0.074 | 0.023 | 0.130 | 0.026 | 2.767 | 0.006 |
|  | VEGCOV + ROCKCOV + VISIBSHORT + VISIBLONG + FEMVISIB3000 + MALEVISIB3000 + FEMVISIB467 + DISTURBAN + DISTTRACK | 0.071 | 0.020 | 0.128 | 0.027 | 2.646 | 0.008 |
|  | VEGCOV + ROCKCOV + VISIBSHORT + VISIBLONG + FEMVISIB3000 + MALEVISIB467 + DISTURBAN | 0.068 | 0.019 | 0.121 | 0.025 | 2.647 | 0.008 |
|  | VEGCOV + ROCKCOV + VISIBSHORT + VISIBLONG + FEMVISIB3000 + MALEVISIB3000 + MALEVISIB467 | 0.067 | 0.019 | 0.119 | 0.025 | 2.651 | 0.008 |
|  | VEGCOV + ROCKCOV + VISIBSHORT + VISIBLONG + FEMVISIB3000 + MALEVISIB3000 + FEMVISIB467 + MALEVISIB467 | 0.065 | 0.016 | 0.118 | 0.025 | 2.527 | 0.011 |
| VISIBLONG | VEGCOV + ROCKCOV + VISIBSHORT + VISIBLONG + FEMVISIB3000 + MALEVISIB3000 + FEMVISIB467 | 0.003 | 3.24x10^-5^ | 0.006 | 0.001 | 1.861 | 0.063 |
|  | VEGCOV + ROCKCOV + VISIBSHORT + VISIBLONG + FEMVISIB3000 + MALEVISIB3000 | 0.003 | -1.64x10^-5^ | 0.006 | 0.001 | 2.071 | 0.038 |
|  | VEGCOV + ROCKCOV + VISIBSHORT + VISIBLONG + FEMVISIB3000 + MALEVISIB3000 + DISTURBAN | 0.003 | 0.0003 | 0.006 | 0.001 | 2.071 | 0.038 |
|  | VEGCOV + ROCKCOV + VISIBSHORT + VISIBLONG + FEMVISIB3000 + MALEVISIB3000 + FEMVISIB467 + DISTURBAN | 0.003 | 0.0003 | 0.006 | 0.002 | 2.110 | 0.035 |
|  | VEGCOV + ROCKCOV + VISIBSHORT + VISIBLONG + FEMVISIB3000 + MALEVISIB3000 + DISTTRACK | 0.003 | -8.77x10^-6^ | 0.006 | 0.001 | 1.835 | 0.066 |
|  | VEGCOV + ROCKCOV + VISIBSHORT + VISIBLONG + FEMVISIB3000 + MALEVISIB3000 + FEMVISIB467 + DISTTRACK | 0.002 | 6.05x10^-5^ | 0.006 | 0.001 | 1.880 | 0.060 |
|  | VEGCOV + ROCKCOV + VISIBSHORT + VISIBLONG + FEMVISIB3000 | 0.002 | 0.0001 | 0.005 | 0.001 | 1.917 | 0.055 |
|  | VEGCOV + ROCKCOV + VISIBSHORT + VISIBLONG + FEMVISIB3000 + MALEVISIB467 | 0.002 | 0.0001 | 0.006 | 0.001 | 1.914 | 0.055 |
|  | VEGCOV + ROCKCOV + VISIBSHORT + VISIBLONG + FEMVISIB3000 + MALEVISIB3000 + DISTURBAN + DISTTRACK | 0.003 | 0.0002 | 0.006 | 0.001 | 2.046 | 0.041 |
|  | VEGCOV + ROCKCOV + VISIBSHORT + VISIBLONG + FEMVISIB3000 + MALEVISIB3000 + FEMVISIB467 + DISTURBAN + DISTTRACK | 0.003 | 0.0003 | 0.006 | 0.001 | 2.092 | 0.036 |
|  | VEGCOV + ROCKCOV + VISIBSHORT + VISIBLONG + FEMVISIB3000 + MALEVISIB467 + DISTURBAN | 0.003 | 0.0005 | 0.006 | 0.001 | 2.206 | 0.027 |
|  | VEGCOV + ROCKCOV + VISIBSHORT + VISIBLONG + FEMVISIB3000 + MALEVISIB3000 + MALEVISIB467 | 0.003 | -1.49x10^-6^ | 0.006 | 0.001 | 1.838 | 0.066 |
|  | VEGCOV + ROCKCOV + VISIBSHORT + VISIBLONG + FEMVISIB3000 + MALEVISIB3000 + FEMVISIB467 + MALEVISIB467 | 0.002 | 4.78x10^-5^ | 0.006 | 0.001 | 1.870 | 0.061 |
|  | VEGCOV + ROCKCOV + VISIBSHORT + VISIBLONG + FEMVISIB3000 + MALEVISIB3000 + FEMVISIB467 | 0.468 | 0.175 | 0.827 | 0.164 | 2.857 | 0.004 |
| FEMVISIB3000 | VEGCOV + ROCKCOV + VISIBSHORT + VISIBLONG + FEMVISIB3000 + MALEVISIB3000 | 0.541 | 0.253 | 0.893 | 0.161 | 3.354 | 0.001 |
|  | VEGCOV + ROCKCOV + VISIBSHORT + VISIBLONG + FEMVISIB3000 + MALEVISIB3000 + DISTURBAN | 0.565 | 0.274 | 0.920 | 0.162 | 3.473 | 0.001 |
|  | VEGCOV + ROCKCOV + VISIBSHORT + VISIBLONG + FEMVISIB3000 + MALEVISIB3000 + FEMVISIB467 + DISTURBAN | 0.491 | 0.198 | 0.849 | 0.163 | 3.008 | 0.003 |
|  | VEGCOV + ROCKCOV + VISIBSHORT + VISIBLONG + FEMVISIB3000 + MALEVISIB3000 + DISTTRACK | 0.5265 | 0.237 | 0.878 | 0.161 | 3.259 | 0.001 |
|  | VEGCOV + ROCKCOV + VISIBSHORT + VISIBLONG + FEMVISIB3000 + MALEVISIB3000 + FEMVISIB467 + DISTTRACK | 0.545 | 0.263 | 0.895 | 0.159 | 3.422 | 0.001 |
|  | VEGCOV + ROCKCOV + VISIBSHORT + VISIBLONG + FEMVISIB3000 | 0.549 | 0.265 | 0.897 | 0.159 | 3.446 | <0.001 |
|  | VEGCOV + ROCKCOV + VISIBSHORT + FEMVISIB3000 + MALEVISIB3000 | 0.615 | 0.333 | 0.964 | 0.159 | 3.854 | <0.001 |
|  | VEGCOV + ROCKCOV + VISIBSHORT + VISIBLONG + FEMVISIB3000 + MALEVISIB467 | 0.559 | 0.274 | 0.909 | 0.160 | 3.489 | <0.001 |
|  | VEGCOV + ROCKCOV + VISIBSHORT + FEMVISIB3000 + MALEVISIB3000 + FEMVISIB467 | 0.557 | 0.275 | 0.909 | 0.159 | 3.487 | <0.001 |
|  | VEGCOV + ROCKCOV + VISIBSHORT + VISIBLONG + FEMVISIB3000 + MALEVISIB3000 + DISTURBAN + DISTTRACK | 0.551 | 0.259 | 0.905 | 0.162 | 3.390 | <0.001 |
|  | VEGCOV + ROCKCOV + VISIBSHORT + VISIBLONG + FEMVISIB3000 + MALEVISIB3000 + FEMVISIB467 + DISTURBAN + DISTTRACK | 0.479 | 0.185 | 0.837 | 0.163 | 2.934 | 0.003 |
|  | VEGCOV + ROCKCOV + VISIBSHORT + VISIBLONG + FEMVISIB3000 + MALEVISIB467 + DISTURBAN | 0.583 | 0.296 | 0.936 | 0.161 | 3.624 | <0.001 |
|  | VEGCOV + ROCKCOV + VISIBSHORT + VISIBLONG + FEMVISIB3000 + MALEVISIB3000 + FEMVISIB467 + MALEVISIB467 | 0.546 | 0.257 | 0.900 | 0.162 | 3.372 | <0.001 |
|  | VEGCOV + ROCKCOV + VISIBSHORT + VISIBLONG + FEMVISIB3000 + MALEVISIB3000 + FEMVISIB467 | 0.113 | 0.009 | 0.234 | 0.057 | 2.000 | 0.046 |
|  | VEGCOV + ROCKCOV + VISIBSHORT + VISIBLONG + FEMVISIB3000 + MALEVISIB3000 | 0.102 | 0.001 | 0.221 | 0.056 | 1.822 | 0.069 |
| MALEVISIB3000 | VEGCOV + ROCKCOV + VISIBSHORT + VISIBLONG + FEMVISIB3000 + MALEVISIB3000 + DISTURBAN | 0.106 | 0.001 | 0.225 | 0.056 | 1.865 | 0.062 |
|  | VEGCOV + ROCKCOV + VISIBSHORT + VISIBLONG + FEMVISIB3000 + MALEVISIB3000 + FEMVISIB467 + DISTURBAN | 0.117 | 0.011 | 0.238 | 0.057 | 2.049 | 0.040 |
|  | VEGCOV + ROCKCOV + VISIBSHORT + VISIBLONG + FEMVISIB3000 + MALEVISIB3000 + DISTTRACK | 0.102 | 0.0007 | 0.219 | 0.055 | 1.830 | 0.067 |
|  | VEGCOV + ROCKCOV + VISIBSHORT + VISIBLONG + FEMVISIB3000 + MALEVISIB3000 + FEMVISIB467 + DISTTRACK | 0.112 | 0.0129 | 0.226 | 0.053 | 2.084 | 0.037 |
|  | VEGCOV + ROCKCOV + VISIBSHORT + FEMVISIB3000 + MALEVISIB3000 | 0.101 | 0.003 | 0.214 | 0.053 | 1.908 | 0.056 |
|  | VEGCOV + ROCKCOV + VISIBSHORT + VISIBLONG + FEMVISIB3000 + MALEVISIB467 |  |  |  |  |  |  |
|  | VEGCOV + ROCKCOV + VISIBSHORT + FEMVISIB3000 + MALEVISIB3000 + FEMVISIB467 | 0.112 | 0.012 | 0.227 | 0.054 | 2.080 | 0.037 |
|  | VEGCOV + ROCKCOV + VISIBSHORT + VISIBLONG + FEMVISIB3000 + MALEVISIB3000 + DISTURBAN + DISTTRACK | 0.105 | 0.001 | 0.223 | 0.056 | 1.865 | 0.062 |
|  | VEGCOV + ROCKCOV + VISIBSHORT + VISIBLONG + FEMVISIB3000 + MALEVISIB3000 + FEMVISIB467 + DISTURBAN + DISTTRACK | 0.117 | 0.011 | 0.237 | 0.057 | 2.046 | 0.040 |
|  | VEGCOV + ROCKCOV + VISIBSHORT + VISIBLONG + FEMVISIB3000 + MALEVISIB3000 + MALEVISIB467 | 0.087 | -0.035 | 0.225 | 0.065 | 1.330 | 0.183 |
|  | VEGCOV + ROCKCOV + VISIBSHORT + VISIBLONG + FEMVISIB3000 + MALEVISIB3000 + FEMVISIB467 + MALEVISIB467 | 0.097 | -0.025 | 0.237 | 0.065 | 1.488 | 0.136 |
| FEMVISIB467 | VEGCOV + ROCKCOV + VISIBSHORT + VISIBLONG + FEMVISIB3000 + MALEVISIB3000 + FEMVISIB467 | 1.189 | -0.393 | 2.517 | 0.740 | 1.606 | 0.108 |
|  | VEGCOV + ROCKCOV + VISIBSHORT + VISIBLONG + FEMVISIB3000 + MALEVISIB3000 + FEMVISIB467 + DISTURBAN | 1.197 | -0.385 | 2.521 | 0.734 | 1.631 | 0.102 |
|  | VEGCOV + ROCKCOV + VISIBSHORT + VISIBLONG + FEMVISIB3000 + MALEVISIB3000 + FEMVISIB467 + DISTTRACK | 1.224 | -0.481 | 2.574 | 0.768 | 1.593 | 0.111 |
|  | VEGCOV + ROCKCOV + VISIBSHORT + FEMVISIB3000 + MALEVISIB3000 + FEMVISIB467 | 1.217 | -0.472 | 2.573 | 0.767 | 1.585 | 0.112 |
|  | VEGCOV + ROCKCOV + VISIBSHORT + VISIBLONG + FEMVISIB3000 + MALEVISIB3000 + FEMVISIB467 + DISTURBAN + DISTTRACK | 1.208 | -0.388 | 2.527 | 0.734 | 1.645 | 0.099 |
|  | VEGCOV + ROCKCOV + VISIBSHORT + VISIBLONG + FEMVISIB3000 + MALEVISIB3000 + FEMVISIB467 + MALEVISIB467 | 1.180 | -0.387 | 2.506 | 0.732 | 1.611 | 0.107 |
| MALEVISIB467 | VEGCOV + ROCKCOV + VISIBSHORT + VISIBLONG + FEMVISIB3000 + MALEVISIB467 | 0.519 | -0.188 | 1.267 | 0.368 | 1.407 | 0.159 |
|  | VEGCOV + ROCKCOV + VISIBSHORT + VISIBLONG + FEMVISIB3000 + MALEVISIB3000 + MALEVISIB467 | 0.588 | -0.134 | 1.353 | 0.376 | 1.560 | 0.118 |
|  | VEGCOV + ROCKCOV + VISIBSHORT + VISIBLONG + FEMVISIB3000 + MALEVISIB3000 + FEMVISIB467 + MALEVISIB467 | 0.190 | -0.658 | 1.064 | 0.435 | 0.439 | 0.660 |
| DISTURBAN | VEGCOV + ROCKCOV + VISIBSHORT + VISIBLONG + FEMVISIB3000 + MALEVISIB3000 + DISTURBAN | -0.0004 | -0.001 | 0.0002 | 0.0004 | -1.273 | 0.203 |
|  | VEGCOV + ROCKCOV + VISIBSHORT + VISIBLONG + FEMVISIB3000 + MALEVISIB3000 + FEMVISIB467 + DISTURBAN | -0.0004 | -0.001 | 0.0002 | 0.0004 | -1.286 | 0.198 |
|  | VEGCOV + ROCKCOV + VISIBSHORT + VISIBLONG + FEMVISIB3000 + MALEVISIB3000 + DISTURBAN + DISTTRACK | -0.0004 | -0.001 | 0.0002 | 0.0003 | -1.147 | 0.251 |
|  | VEGCOV + ROCKCOV + VISIBSHORT + VISIBLONG + FEMVISIB3000 + MALEVISIB3000 + FEMVISIB467 + DISTURBAN + DISTTRACK | -0.0004 | -0.001 | 0.0002 | 0.0003 | -1.146 | 0.251 |
|  | VEGCOV + ROCKCOV + VISIBSHORT + VISIBLONG + FEMVISIB3000 + MALEVISIB467 + DISTURBAN | -0.0005 | -0.001 | 0.0002 | 0.0003 | -1.381 | 0.167 |
|  | VEGCOV + ROCKCOV + VISIBSHORT + VISIBLONG + FEMVISIB3000 + MALEVISIB3000 + DISTTRACK | 0.001 | -0.001 | 0.003 | 0.001 | 1.098 | 0.272 |
|  | VEGCOV + ROCKCOV + VISIBSHORT + VISIBLONG + FEMVISIB3000 + MALEVISIB3000 + FEMVISIB467 + DISTTRACK | 0.001 | -0.001 | 0.003 | 0.001 | 1.074 | 0.282 |
| DISTTRACK | VEGCOV + ROCKCOV + VISIBSHORT + VISIBLONG + FEMVISIB3000 + MALEVISIB3000 + DISTURBAN + DISTTRACK | 0.001 | -0.001 | 0.0033 | 0.001 | 0.941 | 0.346 |
|  | VEGCOV + ROCKCOV + VISIBSHORT + VISIBLONG + FEMVISIB3000 + MALEVISIB3000 + FEMVISIB467 + DISTURBAN + DISTTRACK | 0.001 | -0.001 | 0.0033 | 0.001 | 0.941 | 0.346 |

| **Predictor** | **∑** | **b** | **SE** | **Z** | **P** | **Lower CI** | **Upper CI** |
| --- | --- | --- | --- | --- | --- | --- | --- |
| **VEGCOV** | 1 | -0.179 | 0.040 | 4.439 | <0.001 | -0.257 | -0.100 |
| **ROCKCOV** | 1 | -0.129 | 0.043 | 2.936 | 0.003 | -0.221 | -0.044 |
| **VISIBSHORT** | 1 | 0.071 | 0.026 | 2.638 | 0.008 | 0.019 | 0.127 |
| **FEMVISIB3000** | 1 | 0.527 | 0.169 | 3.115 | 0.002 | 0.202 | 0.871 |
| **VISIBLONG** | 0.92 | 0.003 | 0.002 | 1.558 | 0.119 | -9.147x10^6^ | 0.006 |
| **MALEVISIB3000** | 0.91 | 0.091 | 0.066 | 1.354 | 0.162 | -0.012 | 0.221 |
| **FEMVISIB467** | 0.59 | 0.483 | 0.755 | 0.640 | 0.522 | -0.351 | 2.624 |
| **DISTURBAN** | 0.34 | -0.0001 | 0.0003 | 0.484 | 0.628 | -0.001 | 0.0003 |
| **DISTTRACK** | 0.25 | 0.0003 | 0.001 | 0.368 | 0.713 | -0.001 | 0.003 |
| **MALEVISIB467** | 0.18 | 0.068 | 0.236 | 0.285 | 0.775 | -0.508 | 1.250 |
| **DISTBUILD** | 0.039 | 4.302x10^-6^ | 0.0003 | 0.034 | 0.972 | -0.001 | 0.002 |

**Supplementary Table S5**. Model-averaged estimates of all display site predictor variables selected in models, independently of the ΔAICc value. Values given indicate the relative importance (∑, sum of Akaike weights of the models in which the predictor was present), regression coefficients (b), standard errors (SE), p-values, Z-values (Z) and 95% coefficient intervals for b (CI). See definitions of variables in Table 3
